# Supplementary material for: Discovering Classical Spin Liquids by Topological Search of High Symmetry Nets
Source: ACS Cent Sci. 2024 Sep 11;10(10):1821–8. doi: 10.1021/acscentsci.4c01020 (PMC11503497; doi:10.1021/acscentsci.4c01020)
Supplement: Supplementary file 1 — oc4c01020_si_001.pdf [file oc4c01020_si_001.pdf]

Discovering classical spin liquids by topological search of high symmetry nets: Supporting Information

Joseph A. M. Paddison *[a] and Matthew J. Cliffe †[b]

[a]Neutron Scattering Division, Oak Ridge National Laboratory, Oak Ridge, Tennessee 37831, USA
[b]School of Chemistry, University Park, Nottingham, NG7 2RD, United Kingdom

July 25, 2024

*paddisonja@ornl.gov
†matthew.cliffe@nottingham.ac.uk

Table S1: Complete list of nets investigated.

label	z	Bipartite	space group
**acs**	6	Y	$P6_3/mmc$
**ana**	4	Y	$Ia\bar{3}d$
**bcu**	8	Y	$Im\bar{3}m$
**bcs**	6	Y	$Ia\bar{3}d^*$
**crs**	6	N	$Fd\bar{3}m$
**dia**	4	Y	$Fd\bar{3}m$
**fcu**	12	N	$Fm\bar{3}m$
**hxg**	6	Y	$Pn\bar{3}m^*$
**lcs**	4	Y	$Ia\bar{3}d$
**lcv**	4	N	$I4_132$
**lcx**	8	N	$Pm\bar{3}n^\dagger$
**lcy**	6	N	$P4_132$
**lvt**	4	Y	$I4_1/amd$
**nbo**	4	Y	$Im\bar{3}m$
**pcu**	6	Y	$Pm\bar{3}m$
**qtz**	4	Y	$P6_222$
**reo**	8	N	$Pm\bar{3}m$
**rhr**	4	Y	$Im\bar{3}m$
**sod**	4	Y	$Im\bar{3}m$
**srs**	3	Y	$I4_132$
**thp**	8	N	$I\bar{4}3d$

$*$ cannot use distance criterion to define net from vertices; † not a nearest-neighbor net.

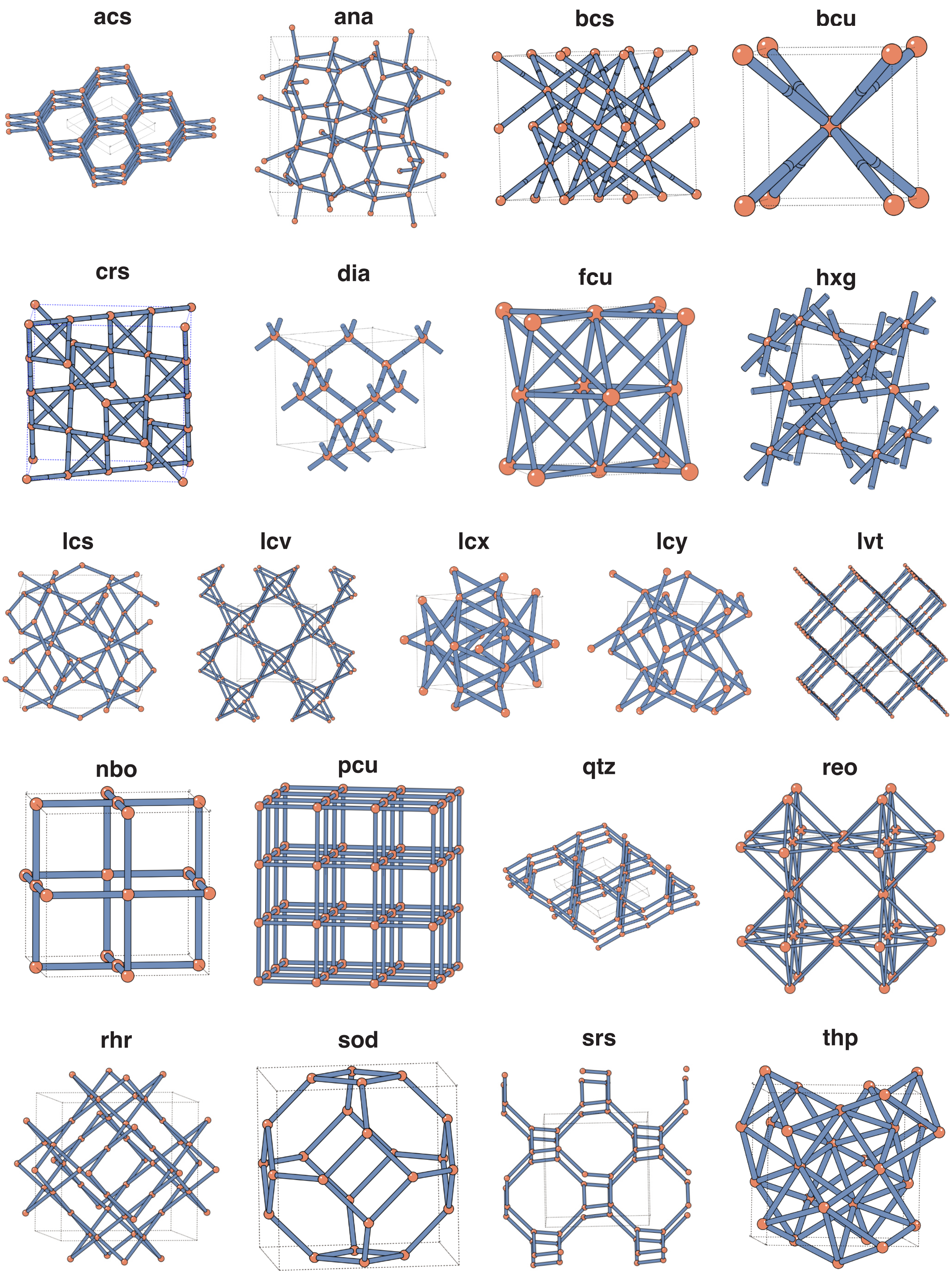

Figure S1: Summary of all edge- and vertex-transitive nets.

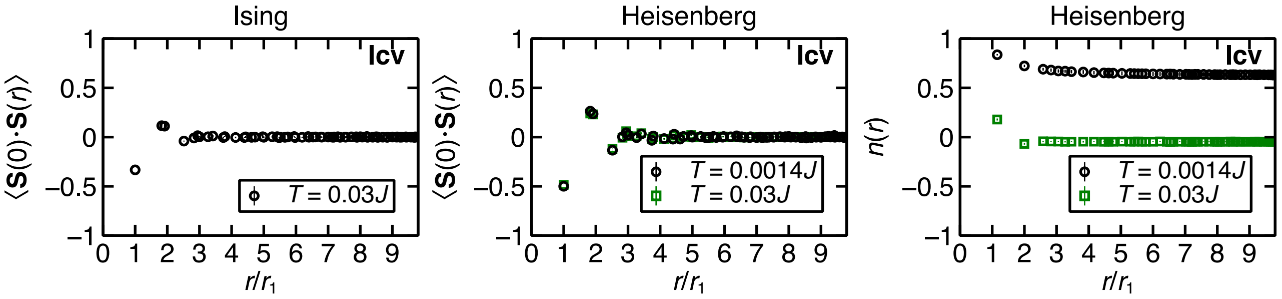

Figure S2: Radial spin-pair correlation function $\langle \mathbf{S}(0) \cdot \mathbf{S}(r) \rangle$ and nematic correlation function $n(r) = \frac{3}{2}\langle [\mathbf{n}(0) \cdot \mathbf{n}(r)]^2 \rangle - \frac{1}{2}$ for antiferromagnetic Heisenberg models on the **lcv** net. Temperatures are labelled in each panel. For each triangular plaquette $\mathbf{n} = \frac{2}{3\sqrt{3}}(\mathbf{S}_1 \times \mathbf{S}_2 + \mathbf{S}_2 \times \mathbf{S}_3 + \mathbf{S}_3 \times \mathbf{S}_1)$. In collinear states, $\langle \mathbf{S}(0) \cdot \mathbf{S}(r) \rangle$ takes the value 1 for ferromagnetic alignment and -1 for antiferromagnetic alignment. In coplanar states, $n(r) = 1$. For the Heisenberg model, coplanar order exists at $T = 0.0014J$ but not at $T = 0.03J$. Results are obtained from $6 \times 6 \times 6$ supercells.

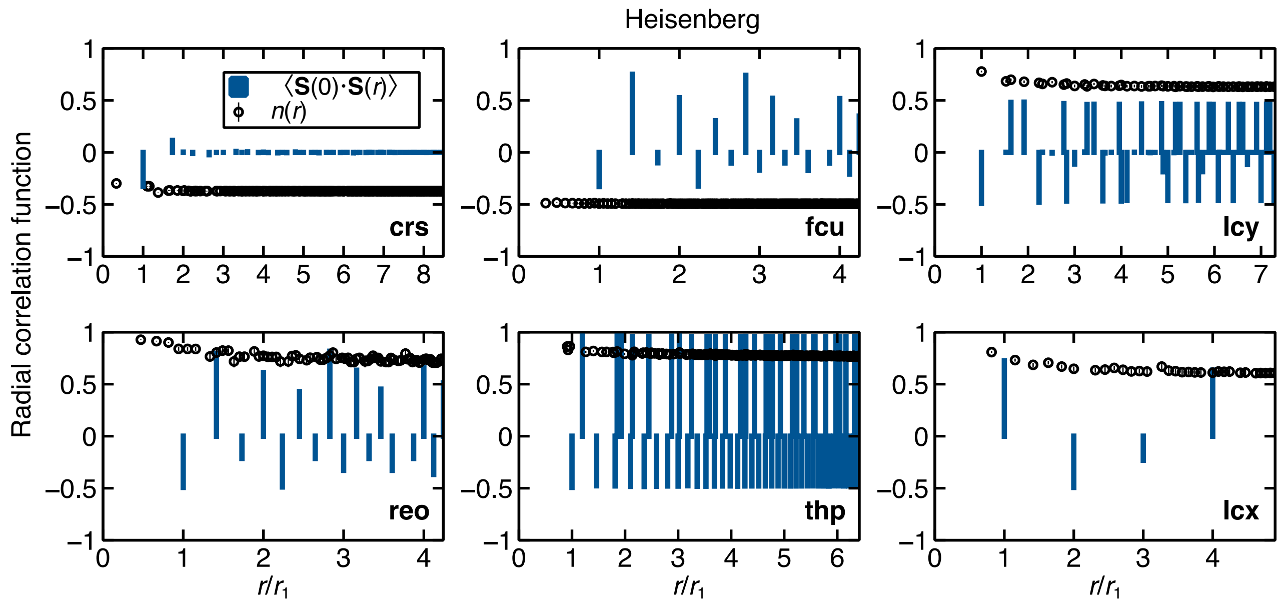

Figure S3: Radial spin-pair correlation function $\langle \mathbf{S}(0) \cdot \mathbf{S}(r) \rangle$ and nematic correlation function $n(r) = \frac{3}{2}\langle [\mathbf{n}(0) \cdot \mathbf{n}(r)]^2 \rangle - \frac{1}{2}$ for antiferromagnetic Heisenberg models on different nets (labelled in the figure panels). For each triangular plaquette $\mathbf{n} = \frac{2}{3\sqrt{3}}(\mathbf{S}_1 \times \mathbf{S}_2 + \mathbf{S}_2 \times \mathbf{S}_3 + \mathbf{S}_3 \times \mathbf{S}_1)$. In collinear states, $\langle \mathbf{S}(0) \cdot \mathbf{S}(r) \rangle$ takes the value 1 for ferromagnetic alignment and -1 for antiferromagnetic alignment. In coplanar states, $n(r) = 1$. Results are at $T = 0.03J$ and are obtained from $6 \times 6 \times 6$ supercells.

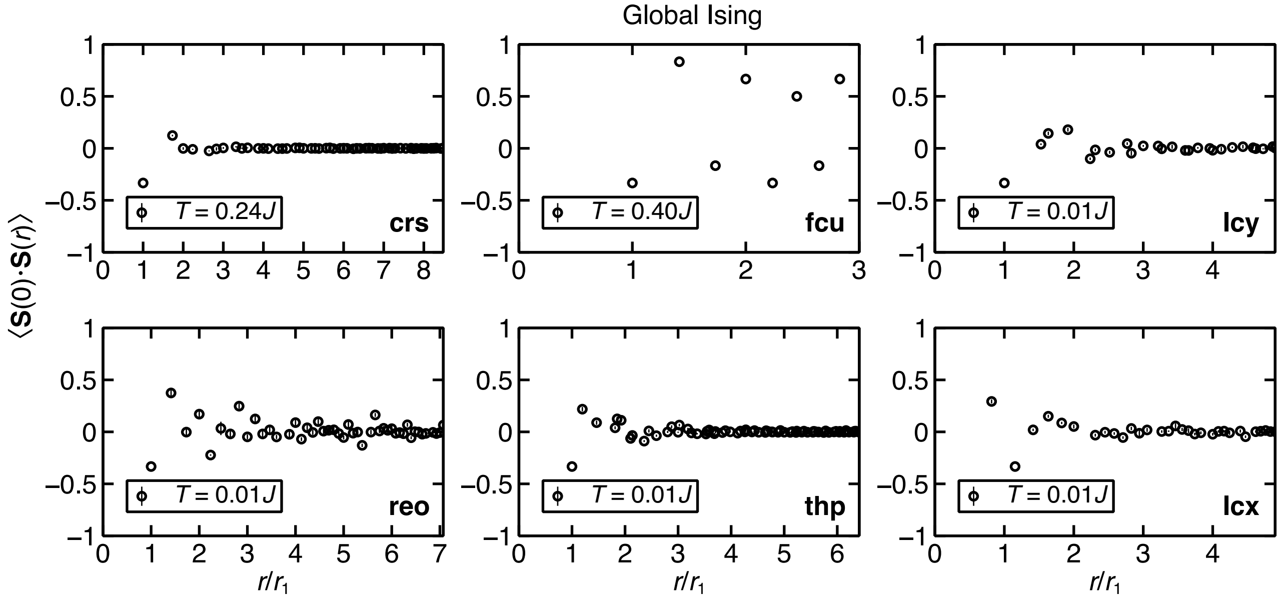

Figure S4: Radial spin-pair correlation function $\langle \mathbf{S}(0) \cdot \mathbf{S}(r) \rangle$ for antiferromagnetic global Ising models on different nets (labelled in the figure panels). Temperatures are labelled in each panel. $\langle \mathbf{S}(0) \cdot \mathbf{S}(r) \rangle$ takes the value 1 for ferromagnetic alignment and -1 for antiferromagnetic alignment. Results are obtained from $6 \times 6 \times 6$ supercells except for **fcu** ($4 \times 4 \times 4$).
